# The impact of *Hafnia alvei* HA4597™ on weight loss and glycaemic control after bariatric surgery — study protocol for a triple-blinded, blocked randomized, 12-month, parallel-group, placebo-controlled clinical trial

**DOI:** 10.1186/s13063-023-07383-0

**Published:** 2023-05-29

**Authors:** Shámila Ismael, Carlos Vaz, Catarina Durão, Marta P. Silvestre, Conceição Calhau, Diana Teixeira, Cláudia Marques

**Affiliations:** 1 Nutrition & Metabolism, CHRC, NOVA Medical School, Faculdade de Ciências Médicas, NMS, FCM, Universidade Nova de Lisboa, Lisbon, Portugal; 2 Nutition & Metabolism, CINTESIS@RISE, NOVA Medical School, Faculdade de Ciências Médicas, NMS, FCM, Universidade Nova de Lisboa, Lisbon, Portugal; 3 Obesity and Metabolic Surgery Unit, Hospital CUF Tejo, Lisbon, Portugal; 4 EPIUnit - Institute of Public Health, Universidade Do Porto, Porto, Portugal; 5 Laboratory for Integrative and Translational Research in Population Health (ITR), Porto, Portugal; 6 Unidade Universitária Lifestyle Medicine José de Mello Saúde By NOVA Medical School, 1169-056 Lisbon, Portugal

**Keywords:** Bariatric surgery, Glycaemic control, *Hafnia alvei* HA4597™, Obesity, Weight loss

## Abstract

**Background:**

Subjects with obesity exhibit changes in gut microbiota composition and function (i.e. dysbiosis) that contribute to metabolic dysfunction, including appetite impairment. Although bariatric surgery is an effective treatment for obesity with a great impact on weight loss, some subjects show weight regain due to increased energy intake after the surgery. This surgery involves gut microbiota changes that promote appetite control, but it seems insufficient to completely restore the obesity-associated dysbiosis — a possible contributor for weight regain. Thus, modulating gut microbiota with probiotics that could improve appetite regulation as a complementary approach to post-operative diet (i.e. *Hafnia alvei* HA4597™), may accentuate post-surgery weight loss and insulin sensitivity.

**Methods:**

This is a protocol of a triple-blinded, blocked-randomized, parallel-group, placebo-controlled clinical trial designed to determine the effect of *Hafnia alvei* HA4597™ supplementation on weight loss and glycaemic control 1 year after bariatric surgery. Patients of Hospital CUF Tejo, Lisbon, that undergo Roux-en-Y gastric bypass are invited to participate in this study. Men and women between 18 and 65 years old, with a BMI ≥ 35 kg/m^2^ and at least one severe obesity-related comorbidity, or with a BMI ≥ 40 kg/m^2^, and who are willing to take 2 capsules of *Hafnia alvei* HA4597™ probiotic supplements (equivalent to 5 × 10^7^ CFU) *vs*. placebo per day for 90 days are included in this study. Assessments are carried out at baseline, 3, 6, 9, and 12 months after the surgery. Loss of weight in excess and glycated haemoglobin are considered primary outcomes. In addition, changes in other metabolic and inflammatory outcomes, gut microbiota composition and metabolites, as well as gastrointestinal quality of life are also being assessed during the trial.

**Discussion:**

The evidence obtained in this study will provide relevant information regarding the profile of the intestinal microbiota of individuals with severe obesity and the identification of the risk/benefit ratio of the use of *Hafnia alvei* HA4597™ as an adjunctive treatment in the maintenance of metabolic and weight control one year after the surgical intervention.

**Trial registration:**

ClinicalTrials.gov NCT05170867. Registered on 28 December 2021.

## Background

Management of obesity stands primarily on achieving an adequate weight loss to reduce its impact on quality of life, morbidity and mortality risk [1, 2]. For patients who are unable to achieve clinically relevant weight loss through therapeutic lifestyle or pharmacological measures, bariatric surgery is considered the most effective intervention for weight loss, prevention of weight regain, and improvement, as well as remission, of many obesity-related comorbidities [3]. Nevertheless, over 20% of all patients do not lose enough weight 1 year after bariatric surgery (< 50% excess body weight) and there is also risk of weight regain after the surgery [4]. Non-adherence to post-surgery dietary recommendations has been reported in 23% of patients, contributing significantly to weight regain [5]. Therefore, identification of new strategies to sustain appetite control to maintain weight loss and metabolic recovery after the surgery is warranted.

It is known that changes in gut microbiota after bariatric surgery play a pivotal role on the beneficial effects related to this surgical intervention, including appetite regulation [6]. In fact, as shown in a clinical study from our group that involved subjects with type 2 diabetes and excess weight [7], gut microbiota changes towards eubiosis preceded glycaemic improvements, demonstrating the important role that the gut microbiome has on metabolic control. Furthermore, previous studies have shown that preoperative gut microbiota composition is a good predictor of bariatric surgery outcomes, including weight loss trajectory [8, 9]. Nonetheless, recent studies indicate that gut microbiota eubiosis is not fully restored after bariatric surgery [6] and that subjects that regained weight have significantly different gut microbiota in comparison to more successful postoperative settings [10]. Hence*,* strengthening the restoration of gut microbiota with probiotics possibly promoting appetite control may be important to improve the metabolic outcomes and weight loss after surgery. Recently, the strain *Hafnia alvei*, a commensal bacterium that belongs to the Hafniaceae family (formerly Enterobacteriaceae), has raised interest regarding its mechanism of action in appetite control [11]. *Hafnia alvei* can synthesize caseinolytic protease B protein (ClpB), which was identified as a conformational antigen mimetic of α-melanocyte-stimulating hormone [11]. ClpB stimulates glucagon-like peptide-1 (GLP-1) and peptide YY secretion by L-cells in the gut and can also stimulate other anorexigenic pathways in the hypothalamus by activating proopiomelanocortin (POMC) neurons [12]. In addition, activation of POMC neurons increases lipolysis and contributes to lowering glucose levels through leptin signalling pathway [11, 13]. Interestingly, gut microbiota of individuals with obesity have low abundance of enterobacterial ClpB [12], which can contribute to appetite dysregulation. Based on these findings, pre-clinical studies were conducted to assess the effect of *Hafnia alvei* as a potential probiotic (*Hafnia alvei* HA4597™) for appetite control and weight management in obese models. Results showed that *Hafnia alvei* HA4597™ supplementation decreased appetite and had anti-obesity and anti-diabetic effects in animal models of obesity [11, 14]. These results were confirmed afterwards in a multicentric, double-blind, randomized, placebo-controlled trial that included 236 overweight subjects, successfully demonstrating the use of *Hafnia alvei* HA4597™ in weight and glycaemic control management [15]. Nevertheless, validation of the effect of *Hafnia alvei* HA4597™ in subjects with obesity is still required, particularly in more severe cases as an adjuvant therapy for bariatric surgery and, possibly, exploring other complementary mechanisms as consequence of gut microbiota modulation, including systemic inflammation and metabolites produced by gut bacteria with relevant metabolic impact. This could contribute to improvement of weight and glycaemic management of subjects with obesity in the long-term and to a better understanding of the role of gut microbiota as therapeutic target in obesity.

## Study objectives

The purpose of this paper is to describe the RESTART project protocol. A study that aims to understand the link between dysbiosis and obesity, assessing the effect of *Hafnia alvei* HA4597™ supplementation as an adjuvant treatment in the context of bariatric surgery for weight loss and glycaemic control. In this regard, the specific aims of this trial are:

Primary aims:

- To evaluate if *Hafnia alvei* HA4597™ supplementation improves the percentage of excess weight loss.
- To assess the impact of *Hafnia alvei* HA4597™ supplementation on glycated haemoglobin (HbA1c).

Secondary aims:

- To evaluate the effect of *Hafnia alvei* HA4597™ supplementation on fasting glucose, HOMA-IR, HOMA-S, HOMA-B, and c-peptide.
- To analyse the effect of *Hafnia alvei* HA4597™ supplementation on gut microbiota composition.
- To analyse the effect of HA4597™ supplementation on gut metabolites: ClpB, short-chain fatty acids (acetate, propionate, and butyrate), trimethylamine N-oxide, indole-3-propionic acid, imidazole propionate.
- To evaluate if *Hafnia alvei* HA4597™ supplementation can improve gastrointestinal quality of life.

## Methods

### Trial design and study setting

**Fig. 1:**
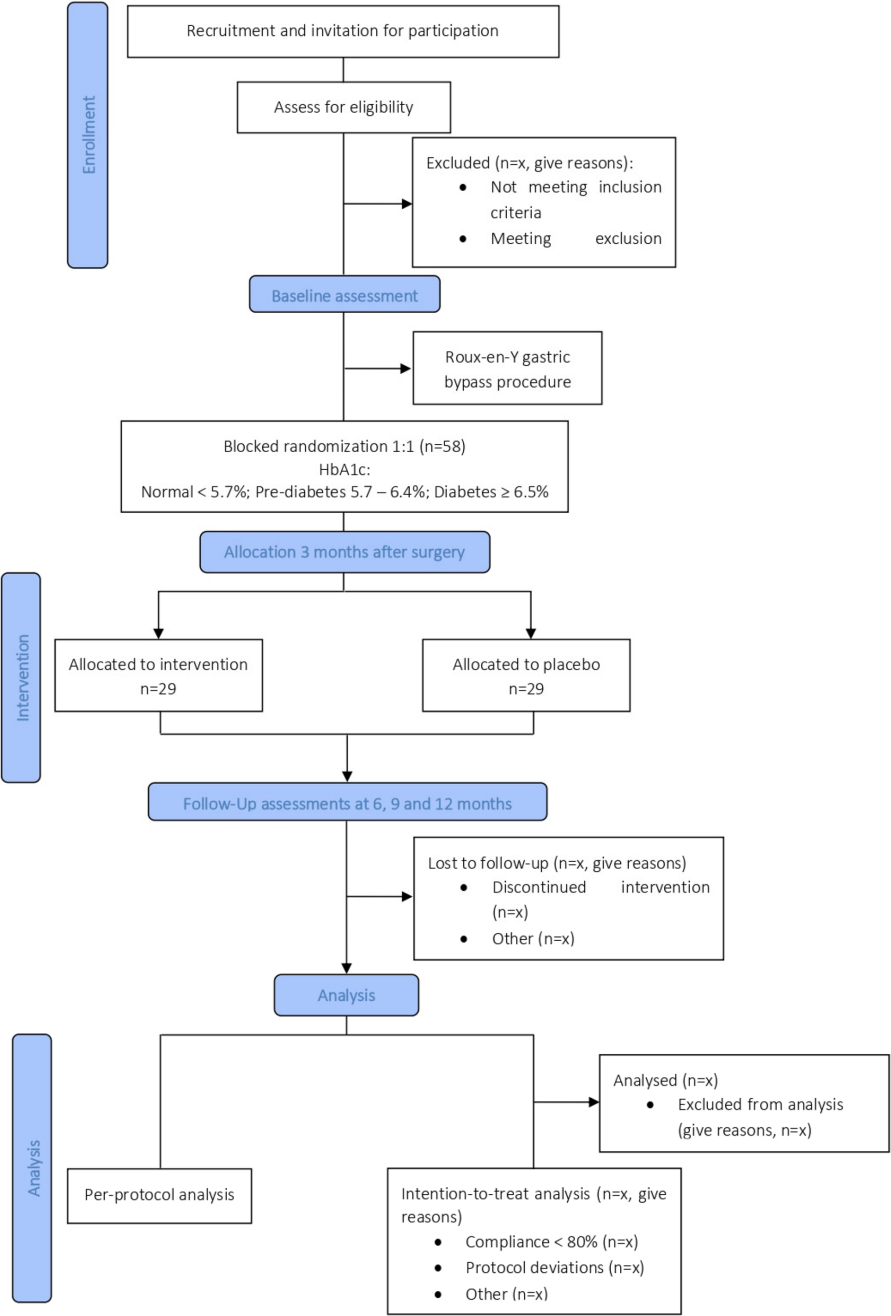
Trial flowchart

This one-year, triple-blinded, blocked-randomized, parallel-group, placebo-controlled clinical trial is designed to determine the effect of *Hafnia alvei* HA4597™ supplementation on weight loss and glycaemic control 1 year after bariatric surgery. The trial is being conducted at Hospital CUF Tejo (Lisboa, Portugal) and samples and data will be analysed at Faculdade de Ciências Médicas, NOVA Medical School, NMS, FCM, Universidade NOVA de Lisboa (Lisboa, Portugal) (NMS, FCM, UNL). The design of the proposed study protocol is shown in Fig. 1.

#### Recruitment and eligibility criteria

Men and women between 18 and 65 years, with a BMI ≥ 35 kg/m^2^ and at least one severe obesity-related complication, or with BMI ≥ 40 kg/m^2^, that will undergo Roux-en-Y gastric bypass for the first time are informed on the trial and invited to participate. Volunteers are recruited after their first surgery appointment and are given at least 1 week to consider their possible participation. Afterwards, if the volunteer decides to participate and agrees to take a probiotic/placebo supplement twice a day for 90 days, the volunteer is asked to give a written informed consent. Subjects are excluded from the study if one or more of the following conditions are met:

- Intake of antibiotics and/or probiotics in the previous 12 weeks
- Weight change > 5% in the previous 12 weeks
- Under corticotherapy or under other anti-inflammatory drugs
- Diagnosis of gastrointestinal disease or other significant illness
- Pregnancy or breastfeeding

#### Randomization, allocation, and blinding

Eligible participants are randomized using block randomization in a 1:1 ratio to either the probiotic or the placebo group. Blocked randomization is stratified according to HbA1c levels obtained at first visit (baseline), following American Diabetes Association guidelines for pre-diabetes and diabetes diagnosis (normal: < 5.7%; pre-diabetes: 5.7–6.4%; diabetes: ≥ 6.5%) [16]. The randomization is performed using the National Cancer Institute clinical trial randomization tool (https://ctrandomization.cancer.gov/). A minimisation method is used on the allocation process to ensure the required balance across the intervention and control groups. Results are sent to an independent researcher responsible for allocation that is not involved in the recruitment nor in the delivery of the intervention. The allocation is performed right before the beginning of the intervention of each group of randomized participants (in blocks), sending the results to a local researcher in a pre-printed Excel worksheet (Microsoft, Redmond, WA, USA). Randomization and allocation of participants are carried out by different researchers.

The study products are packaged and blinded by an outsourced pharmacy. The package appearance of both products is identical; thus, participants and care providers will be blinded to the intervention. All researchers involved in the trial are also blinded to the intervention allocation until the trial is completed. At the end of the study, researchers performing the statistical analysis will divide the participants into the coded groups and will be unaware regarding the intervention allocation. A member of the research group that is not involved in the study nor participates in the investigation meetings is responsible to keep the sealed envelopes with the allocation of each code in a locked drawer. After performing the analyses, the sealed envelope will be opened by the coordinating and principal investigators.

Unblinding will be needed in case of medical event emergencies or serious medical conditions that may occur during the trial whereby the physician needs to know which treatment they have been receiving. However, given the nature of the intervention and placebo, it is unlikely to occur.

### Bariatric surgery

Only subjects that will be submitted to Roux-en-Y gastric bypass are included in this trial. The surgical procedure is performed by the same surgeon according to Hospital CUF Tejo protocol that follows the 2019 bariatric surgery guidelines [3]. Likewise, all the pre and post-surgical appointments, interventions and recommendations will follow Hospital CUF Tejo protocol [17].

### Intervention, compliance, and adverse effects

Participants enrolled in the trial are randomly assigned to receive either probiotic or placebo. The probiotic or placebo is orally taken, twice a day, with one glass of water, approximately 5 min before both breakfast and lunch, for 90 days, between 3 and 6 months after bariatric surgery. Two capsules of the probiotic contain 5 × 10^7^ CFU of *Hafnia alvei* HA4597™, 5 mg of zinc and 20 μg of chromium. The capsules are gastro-resistant which allows the ingredients to resist stomach acidity. A placebo is used as a comparator to determine if the probiotic effect is directly related with the strain (i.e. *Hafnia alvei*). The placebo product is indistinguishable in colour, smell, and taste from the active formulation, and has the same composition but without the live bacteria.

To improve compliance, participants will receive a message every 2 weeks and a call approximately 45 days after starting the intervention to reinforce the intake of the capsules and to register any missing intake on the calendar provided by the research team.

#### Safety monitoring and compliance

Participants are asked to remark on a daily basis in a paper diary any adverse effects during the intervention. Likewise, the research team also monitors all participants throughout the study (through bi-weekly messages and a call) for any adverse events, in particular gastrointestinal symptoms and possible allergies. In case of serious adverse effects, a thorough follow-up will be conducted to investigate the incident and participants will be advised to stop taking the capsules immediately.

To evaluate compliance, participants register the daily intake of the capsules during the 90 days of intervention and are asked to return all the used and unused bottles with the remaining capsules (if any). It is not specified any concomitant care to be permitted or prohibited.

## Outcomes

An overview of the primary and secondary outcomes, descriptive data and timing of assessment is displayed in Table 1.

**Table 1:**
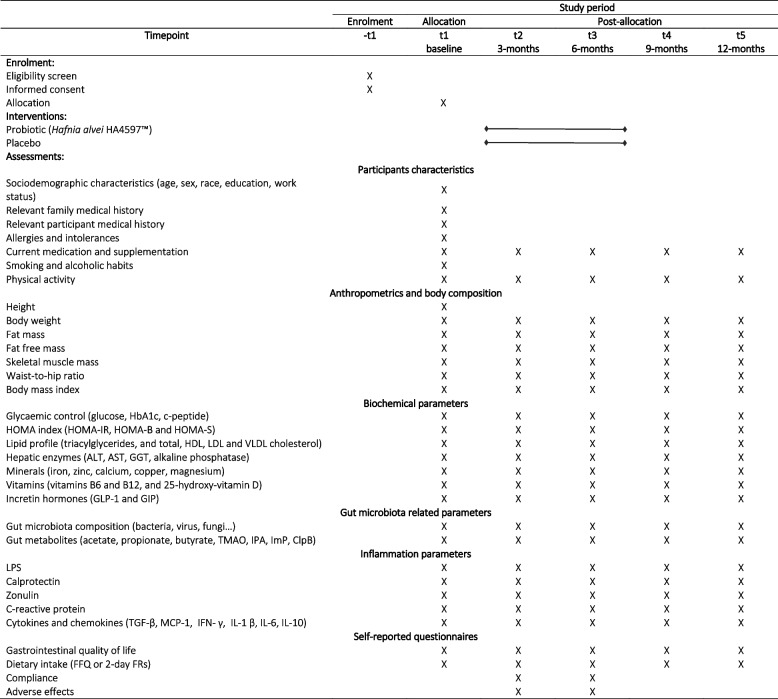
Assessment schedule

*FFQ* Food frequency questionnaire, *FRs* Food records, *HbA1c* Glycated haemoglobin, *HOMA-IR* Homeostatic model assessment of insulin resistance, *HOMA-β* Homeostasis model assessment of β-cell function, *HOMA-S* Homeostasis model assessment of insulin sensitivity, *HDL* High-density lipoprotein cholesterol, *LDL* Low-density lipoprotein cholesterol, *VLDL* Very-low-density lipoprotein cholesterol, *ALT* Alanine aminotransferase, *AST* Aspartate aminotransferase, *GGT* Gamma-glutamyl transferase, *GLP-1* Glucagon-like peptide-1, *GIP* Gastric inhibitory polypeptide, *TMAO* Trimethylamine N-oxide, *IPA* Indole-3- propionic acid, *ImP* Imidazole propionate, *ClpB* Caseinolytic peptidase B protein homolog, *TGF* Transforming growth factor, *MCP* Monocyte chemoattractant protein, *IFN* Interferon, *IL* Interleukin

### Primary outcomes

The primary outcomes are the differences in the number of subjects that will lose at least 50% of excess weight, as well as the number of subjects that will have glycated haemoglobin levels lower than 5.7%, between the probiotic and placebo groups, 1 year after the surgery. The rationale behind the primary outcomes is based on previous studies that have described the effect of *Hafnia alvei* HA4597™. In the preclinical study conducted by Legrand et al. [14], the effect of *Hafnia alvei* HA4597™ supplementation was assessed in two models of obesity, in leptin deficiency *ob/ob* mice and high-fat diet (HFD)-fed obese and overweight mice. Results showed that in both models’ body weight gain was significantly lower by the end of the study (50.1% *ob/ob* and 38.1% HFD), as well as fat gain (38.3% *ob/ob* and 51.9% HFD) [14]. In addition, loss of lean mass was 126.8% lower and food intake reduced 20.8% in the *ob/ob* mice [14]. A second trial was conducted by Lucas et al. [11] using a mice model of obesity characterized by a combination of a HFD-fed obese and a genetic *ob/ob* mice which closely represents the hyperphagia and diet-induced obesity in humans (compulsive eating behaviour combined with hypercaloric diet and functional leptin resistance). The authors observed that *Hafnia alvei* HA4597™ supplementation resulted in 58.1% lower body weight gain, decreased food intake and fat mass gain, and increased lean mass preservation, resulting in an improved lean/fat mass ratio [11]. Moreover, *Hafnia alvei* HA4597™ treatment also improved metabolic outcomes, including glycemia. Basal glucose levels decreased by 1.5-fold compared to standard diet HFD-fed controls [11]. Afterwards, the efficacy of *Hafnia alvei* HA4597™ supplementation was tested in humans [15]. A randomized, placebo-controlled, double-blind trial evaluated the effect of this probiotic (5 × 10^7^ CFU of *Hafnia alvei* HA4597™) on 236 overweight men and women that were following a low-calorie diet [15]. After 12 weeks, significantly more subjects (+ 51%) lost at least 4% of their initial weight comparing to placebo [15]. Furthermore, compared to placebo, subjects in the treatment group had a significant reduction in hip circumference and glycemia, and increased feeling of fullness, which was assessed by a visual analogue scale [15].

Thus, preclinical and clinical results with *Hafnia alvei* HA4597™ are very promising in regard to weight loss and glycaemic levels reduction. Therefore, these outcomes are crucial to validate the effect of *Hafnia alvei* HA4597™ supplementation in an obese population and assess its contribution to the success of bariatric surgery.

### Secondary outcomes

As secondary outcomes, the impact of *Hafnia alvei* HA4597™ supplementation will be assessed towards changes in gut microbiota composition, including diversity, richness, and characterization of microbes at phylum, class, order, family, genus, and species level at baseline, and 3, 6, 9, and 12 months. Alterations in the metabolites produced by gut microbiota that have already demonstrated a protective or a deleterious effect in obesity and type 2 diabetes conditions, namely short-chain fatty acids (acetate, propionate and butyrate), trimethylamine N-oxide, indole-3-propionic acid, imidazole propionate [18–21], will also be determined at the same timepoints. Another important gut metabolite that will be assessed is ClpB, which is specifically produced by *Hafnia alvei* strain and is related to its recognized metabolic effect on appetite regulation [22]. Changes in ClpB levels will be determined before (baseline and 3 months) and after the intervention (6, 9, 12 months).

Additionally, the trial will assess the effect of supplementation on inflammatory markers, including calprotectin, zonulin, c-reactive protein, lipopolysaccharides, transforming growth factor-β, monocyte chemoattractant protein-1, interferon gamma, IL-1 β, IL-6, IL-10, and on other important metabolic outcomes, such as fasting glucose, HOMA-IR, HOMA-S, HOMA-B, and c-peptide, total cholesterol, HDL cholesterol, LDL cholesterol, VLDL cholesterol and triacylglycerides, and vitamins and minerals status (iron, calcium, zinc, copper, magnesium, vitamin B6, vitamin B12, 25-hydroxy-vitamin D) on all visits. GLP-1 and GIP will also be determined at all visits. Lastly, gastrointestinal quality of life impact will be assessed at baseline, and 3, 6, 9, and 12 months.

### Sample collection and follow-up

Eligible participants are assessed five times during the trial: at baseline (visit 1), 3 months (visit 2), 6 months (visit 3), 9 months (visit 4), and 12 months (visit 5) after bariatric surgery. Intervention (i.e. intake of probiotic or placebo) is administered 3 months after the surgery. All data is collected at Hospital CUF Tejo and the specific assessments carried out at each timepoint are identified in Table 1.

Before each visit, participants are contacted and reminded of their follow-up visits and necessary preparations via e-mails and text messages. Participants are asked to prepare for all the visits by following the instructions:

- Fasting for 10–12 h (allowed to drink water 4 h before the visit)
- Having a last meal similar to their usual habits
- Avoiding alcohol and caffeine intake on the previous day
- Avoiding vigorous physical exercise on the previous day

At baseline, participants respond to a questionnaire to provide sociodemographic characteristics, clinical history, and lifestyle practices. Information regarding changes in medication, supplementation and physical activity are continuously gathered throughout the trial through the electronic patient record.

At all visits, a venous blood sample is collected by a nurse/phlebotomist into sterile serum separator tubes and K_2_EDTA tubes. Subsequently, blood samples are centrifuged at 3000 rpm for 10 min at 4 °C, and aliquoted. For incretin hormones aliquot, DPP-IV inhibitor is added to plasma sample to a final concentration of 10 μL/mL of blood. Participants are also instructed to collect their own faecal samples in sterile tubes with RNAlater, provided by the research team. Subjects describe their stool frequency and consistency of evacuation using the Bristol stool scale [23]. All biological samples are stored at − 20 °C (faecal samples) or − 80 °C (plasma and serum samples) until further analysis.

### Anthropometry and body composition

Height is measured to the nearest 0.1 cm with a wall stadiometer (SECA®, Hamburg, Germany), following the Directorate-General for Health protocol [24]. Participants remove their clothes, socks, and any metal objects prior to measurement of body weight and body composition (including percentage of fat mass, fat mass, fat-free mass and skeletal muscle mass) by tetrapolar bioimpedance (Inbody®, model 770, Seoul, Korea). Body mass index is also obtained by the device report and percentage of excess weight loss is calculated as: $\frac{\mathrm{baseline\,weight}\left(\mathrm{kg}\right)-\mathrm{follow\,}-\mathrm{\,up\,weight}\,(kg)}{\mathrm{baseline\,weight}\left(\mathrm{kg}\right)-\mathrm{ideal\,weight\,for\,BMI\,}25\mathrm{\,kg}/\mathrm m^2}\times100$ [25]. Weight and body composition is measured at all visits.

### Gastrointestinal quality of life

Previous studies have shown that the administration of probiotics may improve symptomatic gastrointestinal episodes after gastric bypass surgeries and improve quality of life [26, 27]. Therefore, to assess the gastrointestinal quality of life before and after intervention participants fill out a self-reported questionnaire [Gastrointestinal Quality of Life Index (GIQLI)] at all visits. GIQLI questionnaire includes 36 items divided between five domains: symptoms, physical dysfunction, emotional dysfunctions, social dysfunction, and effect of treatment. The total score is computed as the sum of the response option to each of the 36 items, ranging from 0 (least desirable option) to 4 points (most desirable option). The total score is the sum of item points and ranges from 0 to 144, with higher scores indicating more favourable conditions [28].

### Dietary intake

Dietary intake in the previous 12 months is evaluated by a validated food frequency questionnaire [29] at baseline. Throughout the trial, between visits 2 and 5, subjects complete 2-day food records to describe all foods and fluids consumed in detail including brand names, types of foods and culinary methods. Quantities are described using standard household measures and the information from food labels (where appropriate). A trained dietitian gives the standardised instructions for completing the food diaries and is also responsible for reviewing each record with the participants to clarify errors, omissions, questionable entries, or unclear descriptions. These dietary records will be entered into Food Processor SQL software V.11.1 (ESHA Research, Salem, OR, USA) also by a trained dietitian.

### Glycaemic control, lipid profile, and other blood tests

Routine parameters, including fasting blood glucose, insulin, glycated haemoglobin, c-peptide, triacylglycerides, total cholesterol, high-density lipoprotein (HDL) cholesterol, low-density lipoprotein (LDL) cholesterol, very-low-density lipoprotein (VLDL) cholesterol, alanine transaminase, aspartate transaminase, alkaline phosphatase, gamma-glutamyl transpeptidase, vitamins and minerals (iron, calcium, zinc, copper, magnesium, vitamin B6, vitamin B12, 25-hydroxy-vitamin D) are measured by an outsourced certified medical laboratory. Insulin resistance and sensitivity, and β-cell function are estimated by Homeostatic Model Assessment (HOMA) [30]. GLP-1 (Glucagon Like Peptide 1 (Active) ELISA, Merck®, Darmstadt, Germany) and gastric inhibitory polypeptide (GIP) (Human GIP (total) ELISA, Merck®, Darmstadt, Germany) are measured by ELISA procedure.

### Gut microbiota composition and gut metabolites

Genomic DNA will be extracted and purified from faecal samples using NZY Tissue gDNA Isolation Kit (NZY Tech, Lisboa, Portugal), as described by Marques et al. [31]. Whole-metagenome shotgun sequencing will be performed on the Illumina HiSeq3000 platform to characterize gut microbiota composition as previously described [32, 33]. Gut metabolites [short-chain fatty acids (acetate, propionate, and butyrate), trimethylamine N-oxide, indole-3-propionic acid and imidazole propionate] will be evaluated by targeted metabolomics in serum and faecal samples [34]. ClpB will be assessed in serum and faecal samples by ELISA [Human Caseinolytic peptidase B protein homolog (CLPB) ELISA Kit (MyBioSource, San Diego, CA, USA)], according to manufacturer’s procedures.

### Lipopolysaccharides and inflammatory markers

Quantification of lipopolysaccharide in serum samples will be performed using the Chromo-Limulus Amoebocyte Lysate (Chromo-LAL) reagent (Associates of Cape Cod, Inc., Falmouth, MA, USA), according to manufacturer’s procedures. Faecal calprotectin and zonulin levels will be measured as surrogate markers of intestinal permeability [35] by ELISA kit (IDK ® Calprotectin (stool) and IDK® Zonulin (stool) ELISA kits; Immundiagnostik, Bensheim, Germany). Moreover, c-reactive protein will also be determined in plasma samples as a marker of systemic inflammation by ELISA kit (Human C-Reactive Protein ELISA Kit; Sigma-Aldrich, St. Louis, MO, USA), according to manufacturer’s protocol. Inflammatory mediators including transforming growth factor-β, monocyte chemoattractant protein-1, interferon gamma, IL-1 β, IL-6, and IL-10 will be measured in plasma samples in batch by bead-based LEGENDplex™ analysis (Biolegend, San Diego, CA, USA)] and reactions will be run in duplicate with a BD FACSCantoII flow cytometer (BD-Biosciences San Jose, CA, USA) as described by Castela et al. [36] in a certified medical laboratory (Laboratório de Imunologia e Imunodeficiências Primárias @ NOVA Medical School, Lisboa, Portugal).

## Sample size

Sample size calculations have considered the outcomes: percentage of excess weight loss and fasting blood glucose levels. Based on previous studies with a population similar to this study, the assumed standard deviation was 10.70 for percentage of excess weight loss [37] and 14.00 mg/dL for fasting blood glucose [38]. To detect a difference of 10.48% [39] in excess weight loss and 11.30 mg/dL [40] in fasting glucose between the intervention and placebo group after 1 year of follow-up, it was determined that 44 participants are required to allow for an 80% power and 95% confidence level. Compensating for possible attrition, 30% additional samples will be recruited, representing a total of 58 participants (29 in each arm — probiotic group and placebo group). Considering that Hospital CUF Tejo receives around 80 patients each year to be submitted to bariatric surgery, of these around 70% are Roux-en-Y gastric bypass, assuming that 29% of the subjects will not be eligible to participate [39], it is anticipated that it will take approximately 18 months to recruit this sample size.

## Data management

Data is collected electronically and questionnaires in paper format. The quality of data collection and data entry is maximized through training of field staff in standardised methodology and missing data checks during the study. For data entry, a 5% sample will be validated by another member of the research team to monitor error rates. The data will be inputted into Microsoft Excel programme (Microsoft, Redmond, WA, USA) and exported to SPSS V.27 (IBM SPSS Statistics for Windows, IBM Corporation, Armonk, NY) and GraphPad Prism V.9 (GraphPad Software, La Jolla, CA, USA) for analysis. Verified and validated data will be stored via the cloud on a secure, highly fault-tolerant, storage area network server at NMS, FCM, UNL. All data will be accessible only to the research team. The anonymized participant-level dataset and statistical codes will be made available upon reasonable request. The folders that contain all anonymized research data will be preserved for at least 10 years.

## Statistical analysis

Statistical analysis will be performed using SPSS V.27 (IBM SPSS Statistics for Windows, IBM Corporation, Armonk, NY) and GraphPad Prism V.9 (GraphPad Software, La Jolla, CA, USA).

Participants with low compliance as defined by taking less than 80% of the pills provided by the research team and those lost to follow-up will be excluded from the per-protocol analysis but included in the intention-to-treat analysis. In addition, all participants are strongly advised not to take any probiotic supplements. Participants are asked at each visit about the intake of antibiotics and probiotics supplements, in case of intake in any period of the trial after starting the intervention, participants will also be excluded from the per-protocol analysis but will be included in the intention-to-treat analysis. In case of lost to follow-up, available data from electronic medical records will be collected.

The Kolmogorov–Smirnov test will be used to test the normality of the distribution. Descriptive statistics will be performed, and data will be presented as mean ± standard error of mean (SEM) (for normally distributed continuous variables) or median [interquartile range (IQR)] for nonnormal quantitative data and frequency (percentage) for categorical data. Data will be evaluated for the presence of outliers, violations of normality, and missing data. Major violations of normality will be corrected with an appropriate transformation procedure (i.e. logarithmic transformation for positively skewed and exponential function for negatively skewed data). To analyse the differences in the continuous variables between the intervention and placebo groups, different tests will be used depending on the data distribution. Paired samples *t*-test will be used for normal data and Wilcoxon signed rank test for non-normal data. Categorical variables will be analysed by *X*^2^ test. Correlations between outcomes will be evaluated by Pearson’s chi-square and Spearman correlation test. Relative risks and weighted mean differences will be employed to calculate the dichotomous and continuous data, respectively.

Some prespecified subgroup analysis will be done based on some confounding variables such as age, gender, diagnosis of type 2 diabetes, etc., for detecting heterogeneity in intervention efficacy across the strata of mentioned variables. However, these subsamples will be compared to the main dependent variables at baseline. Potential confounding variables will be evaluated in each model. Regression models will be used to predict which factors at baseline most contribute to excess weight loss 1 year after the surgery.

Differences will be considered statistically significant when *p* < 0.05. Effect sizes (Cohen d test) will also be assessed to decide whether a clinically relevant effect is found (|d|> 0.50 indicates a medium effect size and |d|> 0.80 indicates a large effect size).

## Ethical approval and data collection

The research protocol was approved by the Ethics Committee of NMS|FCM, UNL (120/2021/CEFCM) and Hospital CUF Tejo and has been conducted according to the Good Clinical Practice guidelines (Declaration of Helsinki) and applicable national law. Protocol amendments (if any) will be communicated by principal investigators. Written informed consent is obtained from all participants before any study procedure, and participants are informed regarding the possibility to withdraw from the study at any time without consequences for further treatment and care. All participant information is stored in locked file cabinets in areas with limited access. All laboratory specimens, reports, data collection, process, and administrative forms are identified by a coded ID [identification] number and stored in a separate area from other identifying information only to maintain participant confidentiality. All data is treated in an aggregated way. Collection, coding, processing, storage, and destruction of the data of the participants is the responsibility of the research team. As there is no anticipated harm for study participation, participants consent to not receive any compensation or post-trial care.

The study was registered at ClinicalTrials.gov Database, reference NCT05170867, on 28 December 2021. Regardless of the outcome, the trial results will be disseminated through peer-reviewed publications and presentations at relevant national and international scientific meetings.

## Recruitment status

The patient recruitment is still ongoing, it has started in March 2022 and is expected to be completed by June 2023. To date, 30 subjects have been recruited and 14 were considered eligible and agreed to participate in this trial. The mean age of the eligible participants is 46 years and 50% of subjects are female. At baseline, participants had an average weight of 129.7 kg and an average height of 1.70 m, which corresponds to an average body mass index of 44.9 kg/m^2^ (class III obesity). Moreover, mean glycated haemoglobin was 6.3%, which falls in the reference interval for pre-diabetes.

## Discussion

Gut microbiota modulation after bariatric surgery could be one missing key towards restoring the dysbiotic state reminiscent of an obese-related environment. Re-establishing this imbalance with adequate probiotics may trigger satiety pathways and contribute to anti-inflammatory effects, directly and/or indirectly enhancing bariatric surgery success, including weight loss and glycaemic response management. Past studies that assessed the effect of probiotics on weight loss in humans have been more focused in strains of *Lactobacillus* and *Bifidobacterium* genera [41]. However, the impact of probiotic supplementation after bariatric surgery has shown to be very inconsistent, appearing to be dependent on the type of probiotic, duration of consumption, time of follow-up, and typology of bariatric procedure [26]. This said, well-designed randomized placebo-controlled trials are very scarce, and it is necessary to evaluate the effect of probiotics with strain-specific properties based on a better understanding of the mechanisms of action of commensal bacteria on the host. *Hafnia alvei* HA4597™ could be a potential strain to be used as a probiotic after bariatric surgery based on its anti-obesity, satiety-inducing, and lowering glucose and cholesterol effects, which have been pointed out as consequences of increased levels of ClpB production [11, 14, 15]. Although similar effects have been observed in overweight subjects, unravelling the magnitude of the effect of *Hafnia alvei* HA4597™ in obese individuals, especially as a coadjuvant measure for bariatric surgery on weight loss and glycaemic control is warranted. Adding to this, because subjects with obesity exhibit lower ClpB levels [12], contributing to obesity-related metabolic dysfunction, it is important to assess *Hafnia alvei* HA4597™ supplementation in ClpB production on this target group. Likewise, it is crucial to explore its mechanisms of action in humans, understanding its impact on gut metabolites and systemic inflammation. This could contribute to the establishment of a post-surgery protocol that incorporates gut microbiota as a therapeutic target, which may ultimately contribute to a better success of the surgery and improved quality of life for the patients.

## Risks and benefits

Previous studies have reported no adverse events with the use of *Hafnia alvei* HA4597™ supplementation. Regardless, adverse events are systematically monitored based on the methods and prompt responses will be provided for any incidence and signs of adverse events. During the trial, participants are encouraged and instructed to identify signs of gastrointestinal discomfort, which will be considered an adverse event. On the other hand, by participating in this study, participants will be contributing to scientific knowledge that will result in more successful interventions in the future. In addition, participants may have benefits at an individual level through exposure to compounds with beneficial health properties and due to evaluations and analyses that will be carried out within the scope of this trial that can reflect on a better knowledge of their health condition.
